# Human Gut Microbiota Profiles Related to Mediterranean and West African Diets and Association with *Blastocystis* Subtypes

**DOI:** 10.3390/nu17182950

**Published:** 2025-09-13

**Authors:** Lorenzo Antonetti, Federica Berrilli, Marina Cardellini, Massimo Federici, Rossella D’Alfonso

**Affiliations:** 1 Department of Systems Medicine, University of Rome Tor Vergata, 00133 Rome, Italy; lorenzanto96@gmail.com (L.A.); cardellini@med.uniroma2.it (M.C.); federicm@uniroma2.it (M.F.); 2 Department of Clinical Sciences and Translational Medicine, University of Rome Tor Vergata, 00133 Rome, Italy; 3 Hôpital Générale Saint Louis Orione-Anyama, Anyama 299, Côte d’Ivoire

**Keywords:** gut microbiota, palm oil, olive oil, Mediterranean diet, *Blastocystis* subtype 1,3, Africa

## Abstract

**Background/Objectives**: The effects of geographical origin, alongside age, diet, and drug treatments, on the gut microbiota have not been thoroughly analyzed in African countries. Furthermore, eukaryotic components, including *Blastocystis*, the most common intestinal protozoan worldwide, require further investigation. This study compares the gut microbiota of Italian subjects with that of two African groups to examine the influence of dietary patterns and the effects of *Blastocystis* presence and subtypes. **Methods**: Three cohorts of healthy subjects (Italians residing in Rome, Africans residing in the Côte d’Ivoire, and Africans living in Italy) were compared by sequencing the V3-V4 hypervariable regions of the 16S rDNA gene. Taxa abundance and associations with typical West African and Italian foods were determined using DESeq2. Co-abundant genera were identified with a weighted correlation network analysis (WGCNA). *Blastocystis* subtypes were determined and correlated with the microbial composition in the three groups. **Results**: Distinct microbial taxa were associated with specific foods, including palm oil, Cube Maggi, sunflower oil, and olive oil. A Mediterranean diet consumed for over two years did not alter the abundance of *Faecalibacterium* and *Dorea* in the Africans living in Italy compared with Africans living in Côte d’Ivoire, whereas differences were observed in the abundance of some *Prevotella-*9, *Bacteroides*, and *Lachnospiraceae* OTUs. Significant associations were identified between palm oil and *Subdoligranulum*, Cube Maggi and *Dorea*, sunflower oil and the *Ruminococcus* torques group, and olive oil and *Faecalibacterium*. Concerning *Blastocystis*, alpha and beta diversity analysis showed a significant separation between carriers and non-carriers. **Conclusions**: This study provides the first comparative analysis of gut microbiota composition between individuals from Côte d’Ivoire and Italians focusing on the influence of distinct dietary patterns.

## 1. Introduction

The human gut microbiota is involved in the modulation of numerous functions including the production of energy and metabolites during digestive processes, competition with intestinal pathogens, the promotion of immune homeostasis, and communication with the central nervous system via the gut–brain axis. Given the numerous bacterial functions that take place within it, the gut microbiota can be considered an organ subject to the influence of external and internal factors, such as the environment, diet, lifestyle characteristics, and gastrointestinal health status [1,2,3].

Actinobacteria, Firmicutes, Bacteroidetes, and Proteobacteria are the most abundant phyla in the human gut microbiota whose initial evolution occurs during intrauterine life by transfer from the maternal gut microbiota. Firmicutes and Bacteroidetes are the major phyla in adults and Firmicutes become dominant after age 65 [4].

The microbiota of healthy individuals generally exhibits stability in its primary bacterial constituents, maintaining temporal coherence as long as external factors remain relatively unchanged [5]. Dietary changes may disturb this stability, leading to dynamic fluctuations within the permanent gut microbiota components due to the ability of bacterial metabolic genes to be activated in independent biochemical pathways [6,7]. However, persistent intestinal alterations in the balance between resident beneficial and detrimental bacteria can result in the development of metabolic disorders, despite intrinsic gut microbiota redundancy and resilience [8,9].

According to hygiene theory [10,11], improved sanitary practices have introduced new selective pressures on the gut microbiota of industrialized populations, along with factors contributing to a loss of microbiota diversity, such as the consumption of ultra-processed foods and products from intensive agriculture and livestock farming [12,13].

Comparing populations across diverse geographical areas with varying lifestyles can yield valuable insights into cooperative and competitive bacterial relationships in healthy gut microbiota [14]. This knowledge can contribute to a deeper understanding of the host–bacterial interactions that support eubiosis and may prevent metabolic disorders [15] and diseases not apparently linked to the gastrointestinal tract.

Despite substantial inter-individual variability in the diversity and relative abundance of bacterial species within the gut microbiota, the identification of a clear ‘healthy’ profile remains a major challenge that is still open. Numerous factors (e.g., diet, lifestyle, health status, and environmental conditions) and their impact on the gut microbiota of American, Western European, and non-Western subjects (rural, agricultural, and semi-urban subjects) are increasingly being studied.

In African populations, food preparation and dietary practices may vary across ethnic groups and in local cultures but also as a result of urbanization that induces changes in eating habits [16]. These factors may affect the composition of the intestinal microbiota, the predisposition to non-communicable diseases that in recent years has seemed to increase in the African context [17,18], and also health outcomes associated with migration [19]. To date, approximately 67% of African countries are not included in any clinical studies and disparities persist in understanding the geographic variability of the gut microbiota [20,21,22].

*Blastocystis* is a common and genetically diverse unicellular eukaryote, with at least 40 identified subtypes (STs). Among these, ST1 to ST4 are the most frequently detected in humans [23,24,25]. Although its global prevalence is variable, studies conducted in industrialized countries have primarily focused on symptomatic individuals, and its role within the gut microbiota remains poorly understood [12]. Increasing attention has been directed toward the relationship between gut microbial composition and metabolic and chronic diseases [26,27]. Of particular interest is the potential role of specific subtypes of *Blastocystis* producing metabolites that modulate immune cell activity and contributing to disorder development [28].

Despite its high prevalence in many African regions, studies on the interaction between *Blastocystis* colonization/infection and the gut microbiota in these countries have only recently emerged. Studies in Côte d’Ivoire [29,30] and Algeria [31] investigated the gut microbiota of healthy *Blastocystis* carriers and individuals suspected of intestinal parasitosis, respectively. The findings of both studies revealed favorable differences in the bacterial richness and diversity associated with *Blastocystis* colonization, supporting the hypothesis that it may confer protective effects or, at a minimum, is not associated with gastrointestinal symptoms in humans [25].

This study aims to describe the potential impact of dietary preferences on the intestinal microbiota composition of healthy individuals from three distinct groups: Italians residing in Italy, Africans living in Africa, and Africans residing in Italy. For the present purpose, dietary habits were assessed by a questionnaire to evaluate the influence of consuming foods typical of the Mediterranean and African diets (e.g., olive or palm oil). Identifying dietary factors that promote beneficial microbes and prevent colonization by pathobionts may be crucial for the prevention of diseases associated with dysbiosis.

The second objective of this study is to investigate the presence of *Blastocystis* subtypes, as it is the most widespread parasite in the world, and how it is characterized by genetic diversity influenced by geographical location and lifestyle. Improving the knowledge of the gut microbiota composition in individuals harboring *Blastocystis* is of particular interest, especially considering the associations with diet and host metabolic profiles recently found.

## 2. Materials and Methods

### 2.1. Subject Eligibility

To evaluate dietary differences, we investigated three groups of randomly enrolled adults. The first group (AA) comprised participants living in Anyama, Southern Côte d’Ivoire. The second group (AI) included participants from various African countries who have been residing in the city of Rome, Central Italy, for at least 24 months. The third group (ii) consisted of Italians living in Rome.

Participants were ineligible if they reported chronic conditions such as cancer, autoimmune disease, diabetes, hypertension, or gastrointestinal disorders such as gastroesophageal reflux. They were also excluded if they had taken any dugs that could influence the gut microbiota, particularly antibiotics, proton pump inhibitors, or probiotics within 2 months before stool collection. All subjects had a normal active lifestyle and were not athletes; anthropometric parameters (e.g., weight, BMI) were not recorded. Participants were asked to complete a questionnaire about their food preferences. A questionnaire was developed to identify distinct dietary patterns among participants. It included foods representative of both the Mediterranean and Sub-Saharan African diets. The list of foods for the Mediterranean diet included bread, pasta, yogurt, and olive oil, while the list for the African diet included attieké, foutou, couscous, kabato, plantains, palm oil, and Cube Maggi. The questionnaire also listed common foods such as rice, sunflower oil, eggs, chicken, fish, carrots, and green beans. The portion of the questionnaire focused on African food preferences was validated by doctors at the Hôpital Générale Saint Louis Orione (Anyama, Côte d’Ivoire). Participants of the AI group were restricted to consuming African food no more than once a week.

All participants were assured that the collected data would be analyzed anonymously and that the stool samples would not yield any information about human DNA, but only about intestinal microorganisms. The scientific rationale and protocol for this study were reviewed and approved by the independent ethics committee of Hôpital Générale Saint Louis Orione-Anyama, Anyama, Côte d’Ivoire, on 1 September 2022. The study participants were all adults and their inclusion was voluntary. This research did not involve any invasive procedures that could affect the physical, psychological, or moral well-being of the participants and adhered to the ethical principles established in the Universal Declaration of Human Rights (1948) and the Declaration of Helsinki (1964), including their subsequent revisions.

### 2.2. Sample Collection and DNA Extraction

To collect stool samples, all participants used tubes from Norgen Biotek (3430 Schmon Parkway, Thorold, ON, Canada) containing 2 mL of preservative and inactivating solution. Each sample was anonymized with a code at all stages of the study. All the samples, both those collected in Rome and those from Côte d’Ivoire, were transported at room temperature to the Laboratories of the University of Rome Tor Vergata, Italy, to proceed with DNA extraction. The protocol of QIAamp Fast DNA Stool Mini Kit (Qiagen Ltd., Hilden, Germany) was used following the manufacturer’s instructions and the DNA quantification was performed using a Thermo Scientific™ NanoDrop™ 2000 Spectrophotometer (Thermo Scientific, Waltham, MA, USA).

### 2.3. Blastocystis Carriage Assignment and Subtype Attribution

A 600 bp fragment of the small subunit (SSU) rRNA was tested by end-point PCR for the accurate detection of *Blastocystis* according to the method of Souppard et al. [32] modified using the primers BhRDr and BlastoRD5 as follows. The amplification was performed in 25 μL volume, containing 12.5 μL PCR master mix 2X (Promega Italia S.r.l., Milan, Italy) and 4–3 μL template DNA. The conditions for the amplification were as follows: a step at 95 °C for 2 min, 59 °C for 1 min, and 72 °C for 1 min, followed by 38 cycles at 95 °C for 1 min, 59 °C for 1 min, and 72 °C for 1 min, followed by a final extension at 72 °C for 2 min. PCR amplicons were directly purified and sequenced on both strands by Bio-Fab Research (Rome, Italy). Subtype identification was achieved by comparing the sequences with those deposited in GenBank using the Basic Local Alignment Search Tool (BLAST) by evaluating identity values.

### 2.4. Analysis of Gut Microbiota Composition

#### 2.4.1. 16S Targeted Metagenomic Sequencing

The 3–10 ng/µL of DNA from the human stool samples was sent to the Laboratory of BMR Genomics S.r.l. (Padova, Italy) for bacterial 16S rDNA gene sequencing. The V3–V4 hypervariable regions of the 16S rDNA gene were amplified by a first PCR step using universal 16S primers [33] to generate a sequencing library in Fastq format. The pool of sequencing data was processed using the QIIME2 DADA2 plugin with the denoise-paired option and standard parameters. Taxonomic classification was performed using a Naïve Bayes classifier (sklearn) [34], which was trained on the SILVA database release 138 [35]. OTU counts were then further processed and analyzed using R (Version 4.3.1) and MicrobiomeAnalyst (Version 2.0) [36]. A total read count of 527,385 was discovered in this study with average counts per sample of 15,981.36 (min. to max.: 6048–30,226). We identified a total number of 908 OTUs. After filtering (low count filter: minimum count ≥ 4 and mean abundance in at least 20% of samples and low variance filter: 10% removed based on inter-quantile range), 260 OTUs remained in the study. The generated OTU table was imported and further processed in R using the phyloseq package [37].

#### 2.4.2. Bioinformatics Analyses

For alpha and beta diversity measures, filtered and total sum scaled data has been used. The calculation of alpha diversity indices, including observed, Shannon’s, and Simpson’s, was executed through the ‘estimate richness’ function in the ‘phyloseq’ package version 1.46.0. Beta diversity was assessed using principal coordinate analysis based on Bray–Curtis, Jaccard, and Weighted and Unweighted UniFrac using the “ordinate” function of the “vegan” package. To test the dissimilarities in microbial community structure, a pairwise comparison for the permutational multivariate analysis of variance using distance matrices has been performed using the “pairwise.adonis” function of the pairwise.Adonis R package.

#### 2.4.3. LEfSe Analysis

Linear discriminant analysis Effect Size (LEfSe) [38] was utilized to identify the microbial taxa that characterized the disparities between the two African groups and the Italian group of participants using the LEfSe R package. The raw counts of the filtered OTU table were used as the input. LEfSe parameters were left at their defaults: alpha for ANOVA and Wilcoxon’s tests at 0.05 and a threshold of the logarithmic LDA score at 2.0.

#### 2.4.4. Differential Analysis of OTU Count and Microbial Co-Abundance Network via WGCNA

OTU counts were transformed for DESeq2 with the phyloseq_to_deseq2 function of the phyloseq package and normalized using the Variance Stabilization function of the DESeq2 R package [39]. The Wald test was used to compare the differences in OTUs between the groups. In brief, a generalized linear model is fitted to give the log2 fold change by negative binomial distribution with estimated sample-specific size factors and gene-specific dispersion parameters. The Wald test (nbiomWaldTest of DESeq2) was then used to test for the significance of the log2 fold change. The weighted correlation network analysis (WGCNA) was applied to better characterize gut microbial composition across the AA, AI, and ii groups to identify clusters of co-abundant taxa [40]. A soft thresholding power of 5 was chosen based on the scale-free topology fit index curve. The ‘ward.D2’ method was used to cluster the topological overlap matrix dissimilarity (TOM) of the adjacency matrix and the resulting tree was cut using a hybrid tree cutting algorithm implemented in the cutreeDynamic function using a deepSplit of 2 resulting in 10 co-abundance clusters with no unassigned OTU. To distinguish the clusters, they were arbitrarily assigned a color. The eigengenes in each sample of the resulting clusters were used for further analyses.

#### 2.4.5. Metagenome Function Prediction

Based on the 16S rRNA gene sequencing data, the functional abundances of microbial communities in each co-abundance cluster were predicted using Phylogenetic Investigation of Communities by Reconstruction of Unobserved States 2 (PICRUSt2, Version 2.5.1). PICRUSt2 PWY outputs were analyzed by LEfSe with standard parameters (alpha at 0.05 and LDA score at 2.0) [41].

### 2.5. Subtypes of Blastocystis Carriage and Gut Microbiota Composition

To reveal possible differences in bacteria composition and structure related to *Blastocystis* carriage, the microbiota from all selected individuals was first compared between *Blastocystis*-negative individuals and individuals colonized by *Blastocystis*. Then, the bacterial abundances of *Blastocystis*-negative individuals were compared with positive subjects categorized according to the different *Blastocystis* subtypes. A top 20 analysis was performed to describe the most abundant genera in the *Blastocystis*-negative group and the groups categorized according to the *Blastocystis* subtypes. LEfSe analysis was used to assess compositional differences between groups defined by *Blastocystis* subtyping and the *Blastocystis*-negative group. Finally, associations between the consumption of specific foods and *Blastocystis* carriers and non-carriers subjects were analyzed using a chi-squared test.

### 2.6. Statistical Analysis

Data were analyzed and visualized using R 4.4.3. The statistical analysis was carried out using the Mann–Whitney U test and two-tailed Fisher’s exact test when appropriate. A pairwise Fisher’s exact test was utilized to assess any differences in food preferences between the three groups; those that contribute the most have been identified. We excluded foods that were consumed in equal amounts by all participants. The percentage % in the table indicates the number of subjects who consume the different foods per week. Data are presented as means ± standard deviation (SD). Data normality has been assessed using the Shapiro–Wilk’s test and by analyzing variable distributions in the groups under analysis. A Benjamini–Hochberg-adjusted *p*-value ≤ 0.05 was considered to be statistically significant.

## 3. Results

A total of 35 stool samples (19M/16F) were collected. The participants were classified into the three groups: AA included 11 African individuals residing in Anyama (Côte d’Ivoire), while AI included 9 individuals of African origins residing in Rome. In the AI group, one person was from Madagascar, two were from Cameroon, two were from Togo, one was from Côte d’Ivoire, and one was from Kenya; all had resided in Italy for at least 2 years. The ii group included 15 Italian individuals born in Italy and permanently residing in Rome. Two samples from African women residing in Rome were discarded due to improper collection. The participant characteristics and *Blastocystis* subtypes are reported in Table 1.

No significant differences were found in age and sex among the three groups, as determined by two-way ANOVA with Tukey’s post hoc test and two-sided Fisher’s exact test, respectively (Supplementary Table S1).

All participants completed a form listing their most frequently consumed foods. A pairwise Fisher’s exact test was utilized to assess any differences in food preferences between the three groups. To highlight the dietary differences between groups, we excluded foods consumed equally by all participants and those that contributed the most to group characterization have been identified and included in Table 2.

### 3.1. Blastocystis Subtype Attribution

Overall, 13 out of 35 subjects tested positive for *Blastocystis*: 8/11 in the AA group, 2/7 in the AI group, and 3/15 in the ii group. The distribution of the subtypes in the three groups is indicated in Table 1. *Blastocystis* ST3 was found only within the African AA and AI groups.

### 3.2. Gut Microbiota Composition

**Figure 1:**
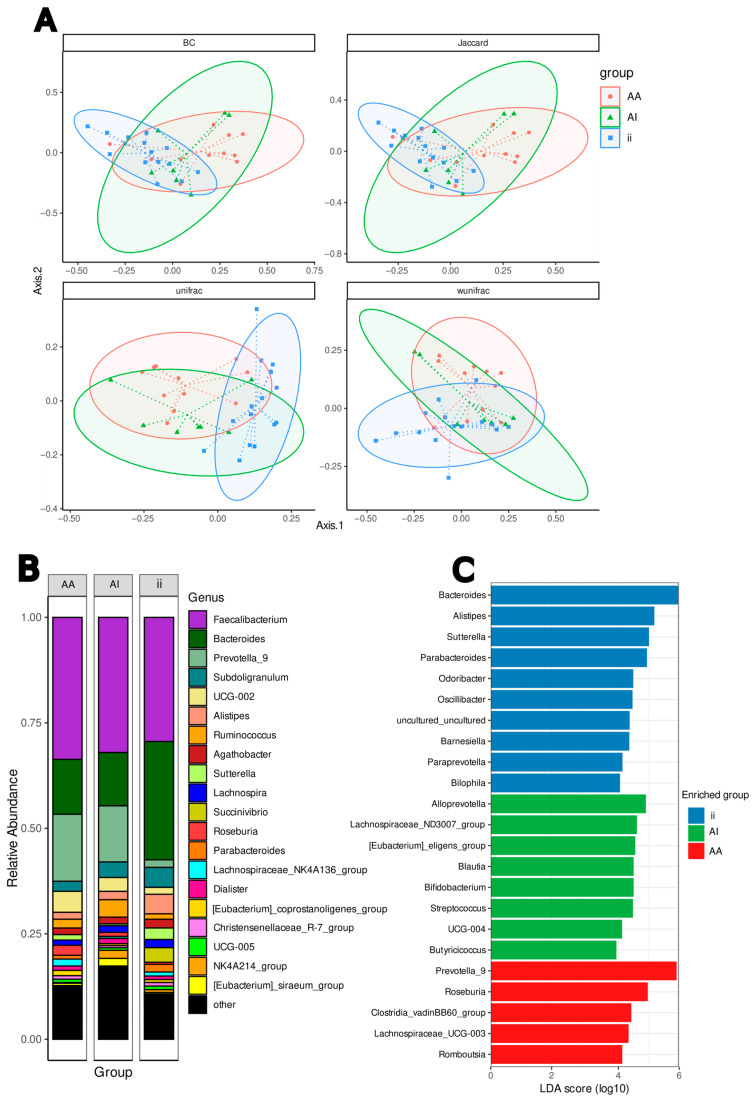
Comparison among African subjects residing in Côte d’Ivoire (AA), African subjects from different countries (AI), and Italian individuals residing in Rome (ii). (**A**) Beta diversity analysis. (**B**) Plot of top 20 relative bacterial abundances at genus level. (**C**) Histogram at genus level of the linear discriminant analysis (LDA scores).

In the comparison between the Ivorian African group (AA), the African group residing in Rome (AI), and the Italian group (ii), alpha diversity did not show significant differences (Figure S1), while beta diversity showed a significant separation between the two African groups and the Italian group (*p* < 0.05) according to the pairwise.adonis test (Figure 1A).

In the analysis of the bacterial abundances of the gut microbiota, the top 20 plot revealed differences at the genus level among the three groups of participants. In detail, the three groups exhibited a similar abundance of *Faecalibacterium* (Ruminococcaceae). Groups AA and AI showed a similar abundance of *Prevotella*-9 (Prevotellaceae family), and *Bacteroides* (Bacteroidaceae family); group ii presented a domination by *Bacteroides* (Bacteroidaceae family) (Figure 1B).

#### 3.2.1. Gut Microbiota and Comparisons of Taxa Abundance

The distinctive microbial taxa of the three groups were compared using the LDA score which confirmed significant differences in the relative abundances of some genera as revealed by the top 20 analysis: *Prevotella*_9 and *Roseburia* in the AA group; Alloprevotella in the AI group; *Bacteroides*, *Alistipes*, *Sutterella*, and *Parabacteroide*s in the ii group (*p* < 0.05). Furthermore, among the minor distinctive taxa, the analysis revealed a significantly higher abundance (*p* < 0.05) in three genera of group AA, six of group AI, and five of group ii, as shown in Figure 1C.

**Figure 2:**
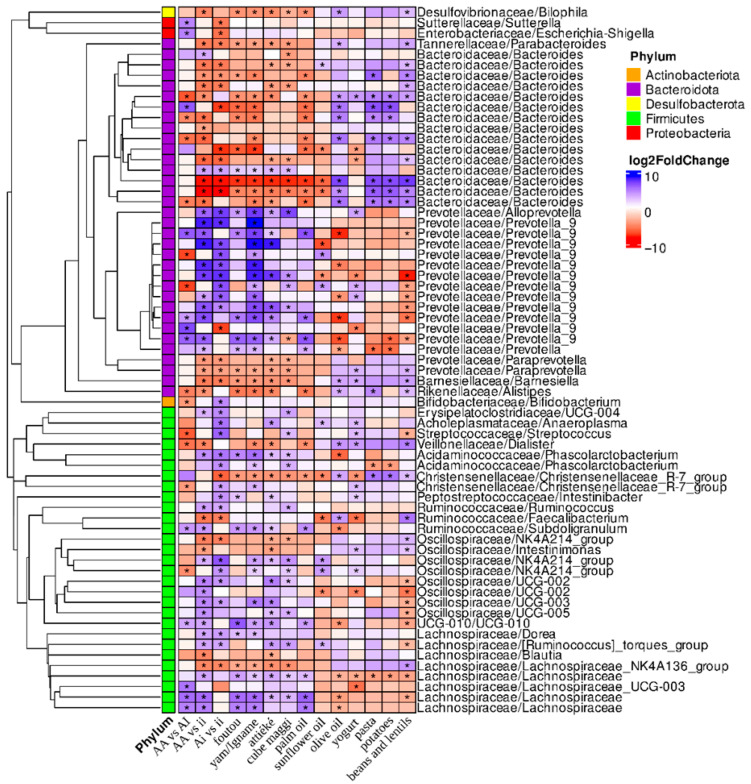
DESeq2 comparative analysis among AA, AI, and ii groups. Association with dietary habits, comparing subjects who consume the food against those who do not. * means *p* < 0.05 in the log2 fold change comparison.

Using a DESeq2 comparative analysis between groups AA and AI, 67 OTUs (Operational Taxonomic Units) were identified as being significantly differentially abundant in at least one comparison (Figure 2).

Within the phylum Bacteroidota, the most represented families are Bacteroidaceae and Prevotellaceae, with several OTUs of *Bateroides* and *Prevotella*_9, respectively; within the phylum Firmicutes the most represented families are Lachnospiraceae and Oscillospiraceae in accordance with the associations obtained by the top 20 relative abundance and LEfSe (Figure 1B,C).

The majority of OTUs of *Prevotella*_9, *Bacteroides*, and *Oscillospiraceae* and OTUs of *Ruminococcus*, *Parabacteroides*, *Alistipes*, *Dialister*, *Faecalibacterium*, and the *Lachnospiraceae* NK4A136 group represented in the top 20 abundance (Figure 1B) showed the same trend in abundances in the AA and AI groups compared with those of group ii according to the DESeq2 comparative analysis (Figure 2). *Alloprevotella*, *Erysipelotrichaceae* UCG-004, *Phascolarctobacterium*, *Oscillospitaceae* UCG-003, UCG-010, *Dorea*, the *Ruminococcus* torques group, *Bilophila*, *Paraprevotella*, and *Barnesiella*, among the minor taxa, showed the same trend in abundances in the AA and AI groups compared with those of group ii.

The impact of the foods (Table 2) in the typical diets of group ii (olive oil, pasta, potatoes, beans, lentils, yogurt, sunflower oil) and groups AI and AA (yam, attiéké, foutou, Cube Maggi, palm oil) was assessed using DESeq2 comparative analysis (Figure 2). Within the phylum Bacteroidota, *Bacteroides* and *Prevotella*_9 OTUs exhibited significant opposing trends between African and Italian participants. In AA and AI subjects, several OTUs of *Bacteroides* were positively associated with pasta, potato, bean, and lentil consumption and negatively associated with foutou, yam, attiéké, and palm oil consumption. The increase in *Prevotella*_9 was positively associated with the consumption of yam, Cube Maggi, or palm oil and negatively with characteristic components of the Mediterranean diet, such as olive oil, pasta, beans, and lentils. Several OTUs of *Paraprevotella*, *Barnesiella*, and *Alistipes* show an increase in the ii group compared with the AA and AI groups, as well as in participants consuming olive oil, pasta, beans, and lentils. On the contrary, *Alistipes* OTUs showed a significant negative association with foutou, attiéké, and palm oil. Within the phylum Firmicutes, the majority of OTUs belonging to *Lachnospiraceae* and *Oscillospiracea* exhibited a significant increase in abundance in the AA and AI compared with the ii group of subjects, except for *Lachnospiraceae*_NK4A136 and *Oscillospiracea* NK4A214 (Figure 2).

Enterobacteriaceae with *Salmonella–Shigella* and *Sutterellaceae* with *Sutterella* do not show differences associated with any food but present a lower abundance in the group AI compared with groups AA and ii.

#### 3.2.2. Analysis of Co-Abundant Microbial Genera Using WGCNA

**Figure 3:**
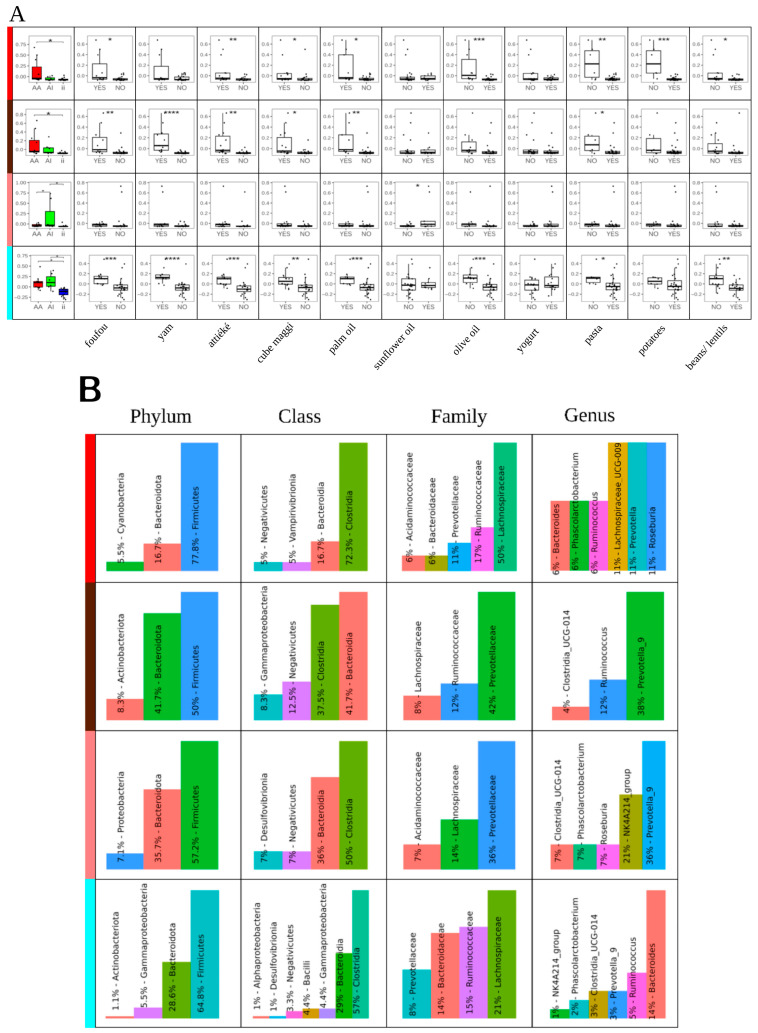

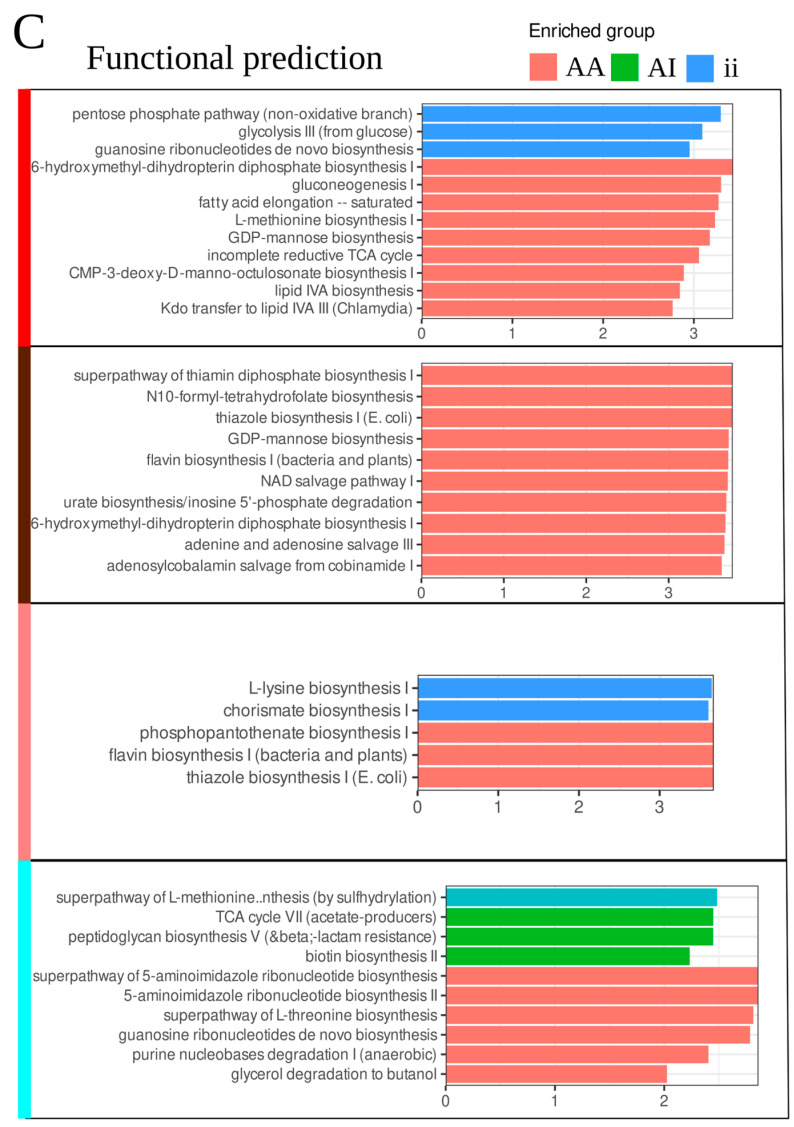
Clusters were color coded (red, brown, pink, turquoise), with data from each cluster plotted on the same line. (**A**) Clustering of OTU counts by weighted correlation network analysis (WGCNA). All four clusters were significantly associated with the residence of subjects in at least one comparison by Benjamini–Hochberg-adjusted two-way ANOVA on the eigengenes. Unpaired two-tailed Student’s *t*-test tested the associations between dietary habits and each significant microbial cluster (*p*-values adjusted using Benjamini–Hochberg method). (**B**) Relative taxonomic composition at phylum, class, family, and genus level of OTUs clustered in a WGCNA cluster associated with AA, AI, ii groups. (**C**) Results of LEfSe analysis on PICRUSt2 functional predictions of OTUs clustered in a WGCNA cluster associated with AA, AI, ii groups. Functional prediction bars are colored by the enriched AA, AI, ii groups. All *p*-values were corrected for multiple testing using Benjamini–Hochberg criterion. **** means *p* < 0.0001, *** means *p* < 0.001, ** means *p* < 0.01, * means *p* < 0.05.

To delve deeper into the microbial composition of all examined subjects, a weighted correlation network analysis using the WGCNA R package was performed to construct a co-abundance network from 16S metagenomic data. A total of 260 OTUs were clustered in 10 co-abundance clusters. Only four distinct microbial clusters were significantly associated with the participants’ place of residence after eigenvalue submission to a two-way ANOVA (Figure 3A). For visual clarity, clusters were arbitrarily color coded: red, brown, pink, and turquoise. The unpaired two-tailed Student’s *t*-test revealed several significant associations between cluster and dietary habits (Figure 3A). For each of the four clusters, the microbial composition at various taxonomic levels and the results of the LEfSe analysis of functional predictions using PICRUSt2 have been reported (Figure 3B). In the red and turquoise clusters, OTUs from the Lachnospiraceae family are the most abundant. However, at the genus level, the red cluster is dominated by *Lachnospiraceae*_UCG-003 and *Roseburia* (Lachnospiraceae family), while the turquoise cluster is characterized by *Bacteroides* (Bacteroidaceae family). In the brown and pink clusters, OTUs from the Prevotellaceae family and *Prevotella*_9 at the genus level are the most abundant (Figure 3B).

Overall, the red and brown clusters showed a significant abundance of OTUs enriched in the AA group and were also positively associated with the consumption of African diet foods. The red cluster results were inversely associated with the consumption of a Mediterranean diet rich in fiber and unsaturated fatty acids and associated with foods such as olive oil, pasta, potatoes, and beans/lentils. The pink cluster showed a major significant abundance of OTUs enriched in the AI group and only a negative association with the consumption of sunflower oil. The turquoise cluster showed that OTUs were less abundant in the ii group than in the AA and AI groups. Food-related associations were positively significant for the consumption of African diet foods and negatively significant for Mediterranean diet foods; only the associations with the consumption of sunflower oil, yogurt, and potatoes were not significant (Figure 3A).

The LEfSe analysis revealed in each cluster a higher number of microbial functional pathways associated with the AA group. In the red cluster, the functional profiles of group ii were enriched in genes associated with the purine biosynthesis and carbohydrate metabolism pathways. Group AA exhibited genes not only related to carbohydrate metabolism but also involved in fatty acid metabolism, cofactor synthesis, and energy production.

Within the brown cluster, ten identified functional pathways were exclusively associated with the AA group. Notably, some of these pathways are implicated in the biosynthesis of essential cofactors for energy production, such as flavins, adenosylcobalamin, and thiamines (Figure 3B). The pink cluster showed in group ii higher capacities for the biosynthesis of L-lysine and aromatic amino acids (chorismite) and in the AA group higher capacities for the biosynthesis of precursors of essential cofactors (flavin, phosphopantothenate) and biologically active compounds (thiazole). Lastly, the turquoise cluster showed a higher abundance of functional profiles originating from the AA group (six) compared with those from the AI (three) and ii groups (one). In detail, group AA showed six microbial pathways related to amino acid and nucleotide metabolism, as well as anaerobic pathways for purine and glycerol degradation. Group AI included three pathways associated with peptidoglycan and biotin synthesis, and energy production. The sole pathway in group ii was linked to L-methionine synthesis.

### 3.3. Gut Microbiota Variation in Blastocystis Carriers

The alpha diversity analysis between *Blastocystis* non-carriers and carriers revealed observed OTUs, Shannon’s, and Simpson’s indices that were significantly different (*p* < 0.05) (Figure S2). According to the Bray, Jaccard, and UniFrac distances, beta diversity showed a significant separation between the two groups (Figure S3).

**Figure 4:**
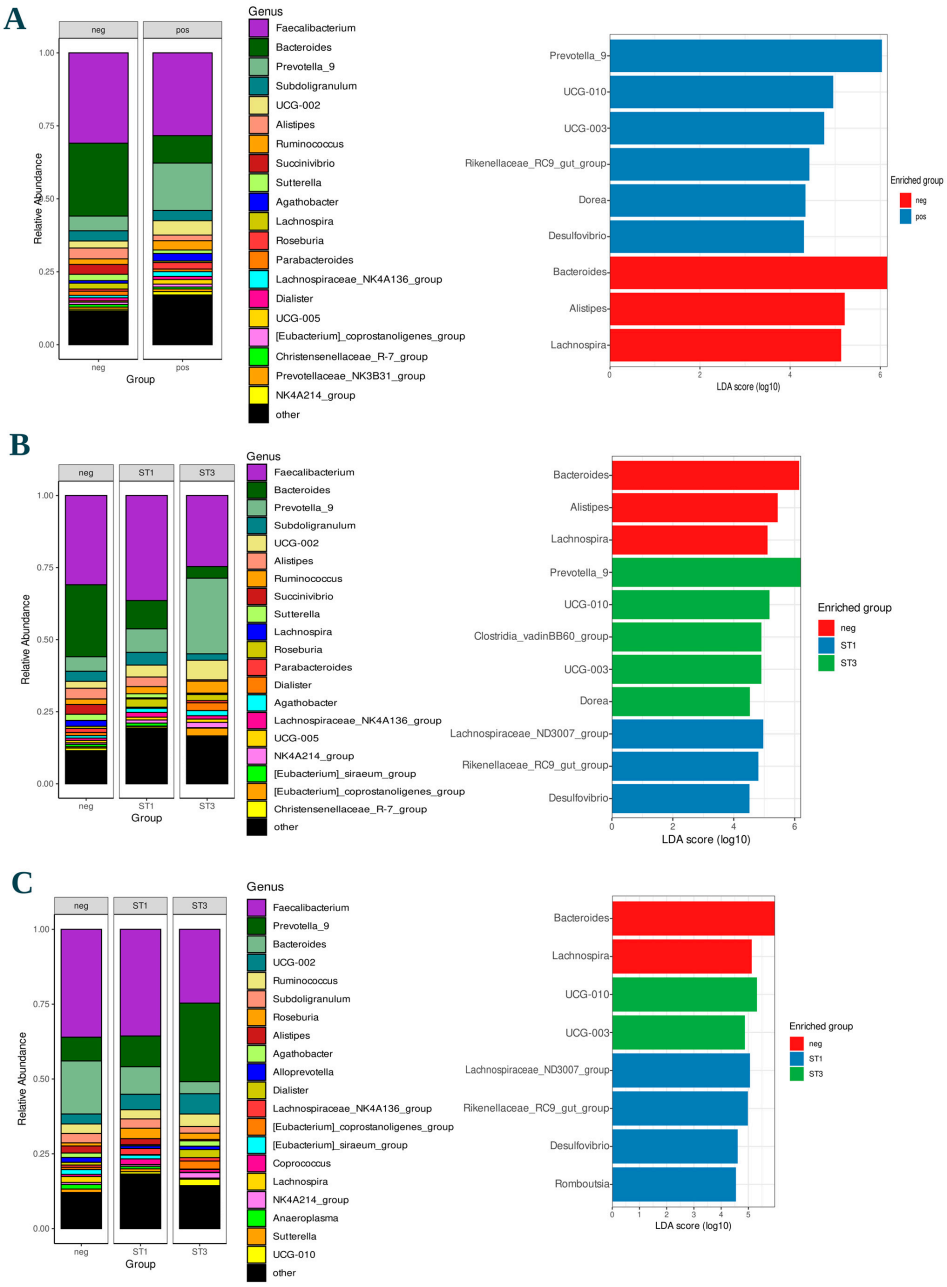
Top 20 most abundant genera composition and LEfSe analysis between (**A**) *Blastocystis* non-carriers and carriers; (**B**) *Blastocystis* ST1 and ST3 subtype carriers versus non-carriers in all subjects; (**C**) *Blastocystis* ST1 and ST3 versus non-carriers in AA and AI groups.

The linear discriminant analysis Effect Size (LEfSe) (Figure 4A) at the genus level showed *Blastocystis* carriers characterized by *Prevotella*_9 and other five minor taxa (*Lachnospiraceae*_UCG-10, *Lachnospiraceae*_UCG-003, *Rikenellaceae*_RC9_gut_group, *Dorea*, *Desulfovibrio*); *Blastocystis* non-carriers were characterized by *Bacteroides*, *Alistipes*, and *Lachnospira*. The same proportion of *Prevotella*_9 and *Bacteroides* in the two groups was shown by the top 20 analysis (Figure 4A).

Due to the limited number of *Blastocystis* subtypes isolated, comparisons were only possible between prevalent ST1 and ST3 subtypes and *Blastocystis* non-carrier subjects. The alpha diversity indices were not significantly different in all comparisons while the Bray, Jaccard, and UniFrac beta diversity indices showed a significant distance between the clusters of non-carriers and ST3 *Blastocystis* carriers (*p* < 0.05) (Figures S4 and S5). According to the linear discriminant analysis Effect Size (LEfSe) at the genus level, differential abundances were identified. Going into detail, *Blastocystis* ST1 carriers were identified by the presence of the *Lachnospiraceae*_ND3007_group, *Rikenellaceae*_RC9_gut_group, and *Desulfovibrio*, among minor taxa. *Blastocystis* ST3 carriers were associated with *Lachnospiraceae*_UCG-10, *Lachnospiraceae*_UCG-03, the *Clostridia*_vadinBB60_group, and *Dorea*, among minor taxa, as well as *Prevotella*_9, among more abundant taxa. In contrast, *Blastocystis* non-carriers were characterized by *Bacteroides*, *Allistipes*, and *Lachnospira* among more abundant taxa. Among *Blastocystis* non-carriers and *Blastocystis* ST1or ST3 carriers, the comparison at the genus level of the top 20 relative bacterial abundances showed the same proportion of *Prevotella*_9, *Bacteroides*, *Allistipes*, and *Lachnospira* (Figure 4B).

Given the low prevalence of *Blastocystis* in Italy, the analysis of intestinal microbiota composition was restricted to the African groups. The variations in bacterial composition associated with *Blastocystis* ST1 and ST3 were analyzed within the AA and AI groups. According to the linear discriminant analysis Effect Size (LEfSe) at the genus level, considering the AA and AI groups combined, the *Lachnospiraceae*_ND3007_group was specifically associated with *Blastocystis* ST1 carriers.

According to the top 20 relative bacterial abundances, *Bacteroides* was confirmed to be more abundant in *Blastocystis* non-carriers and *Prevotella*_9 more abundant in *Blastocystis* ST3 carriers than *Blastocystis* non-carriers and *Blastocystis* ST1 carriers while *Faecalibacterium* showed an opposite trend (Figure 4C). Finally, using the chi-squared test, the consumption of specific African foods, namely foutou, attiéké, and palm oil, was found to be positively associated with *Blastocystis* infection (Figure S6).

## 4. Discussion

Different human cultural traditions introduce factors valuable for studying and understanding host–gut microbiota relationships and microbiota-related diseases [42]. In many African contexts, various ethnic groups with differences in dietary habits coexist in the same area. As a result, socio-cultural factors can make it more complex to study the microbiota within a given geographical area and to establish a univocal definition of intestinal homeostasis and its relationship with digestive diseases [17]. The present comparative study aimed to enhance the understanding of the human gut microbiota by examining three groups: group AA (individuals native to Africa from Côte d’Ivoire), group AI (individuals native to African countries residing in Italy), and group ii (people native to Italy and living in Rome).

The beta diversity divergence observed between the two groups of Africans (AA and AI) and the group of Italians (ii) was expected due to the different geographical origins of the participants [43,44]. The similar abundance of *Faecalibacterium* observed across all three groups could explain the absence of intestinal symptoms supporting the association of this beneficial genus with a healthy gut microbiota (Figure 1B).

The most significantly enriched taxa in the three groups belong to Bacteroidaceae (*Bacteroides*) and Prevotellaceae (*Prevotella*_9 and *Alloprevotella*) (Figure 1C) as shown by Gorvitovskaia [45] in a comparison between American, Western European, and non-Western subjects. Conversely, the abundances of *Prevotella*, *Alistipes*, *Barnesiella*, *Subdoligranulum*, *Ruminococcus*, *Blautia*, and *Dorea* were not entirely consistent with those observed in other comparative analyses across individuals. Discrepancies observed in the compared data can probably be attributed to several factors: the subject selection criteria, which were specific to the aims of the individual studies; the heterogeneity within comparison groups; and variations in microbiota measurements. These factors, along with the cultural traditions of the analyzed groups (and not exclusively their ethnic origins), may account for the observed differences [4,14,16,20,46]. Moreover, higher variability between individuals could be related to greater taxonomic than functional diversity of intestinal bacteria, as suggested by [42].

The native profile in African individuals was only marginally influenced by some rare or abundant taxa, in agreement with Brooks et al. [47]. Indeed, in DeSeq 2 analysis, in both AA and AI groups some OTUs of *Bacteroides*, *Prevotella*_9, *Oscillospiraceae*, and *Lachnospiraceae*, along with *Parabacteroides*, *Alloprevotella*, and *Paraprevotella* (Figure 2) showed a concordant trend compared with group ii (Figure 2). The stability of the intestinal microbiota seems to have partly preserved the profile of the AI group that is common to many African populations, despite the dietary variations during the months of residence in Italy. This partial stability is also confirmed by the WGCNA (Figure 3). In the red clusters, where the OTUs of the AA group are more represented than those of the ii group, *Prevotella* is more abundant than *Bacteroides*. In the turquoise cluster, consisting of the contribution of the OTUs of all of the groups, *Bacteroides* is the most abundant genus; in the other two clusters, *Prevotella*_9 prevails.

Foutou, yam, attiéké, and palm oil are still very widespread in urban areas of West Africa, as in rural and agricultural communities. The consumption of these unprocessed or minimally processed foods was associated with the co-exclusion of *Prevotella*_9 and *Bacteroides* (phylum Bacteroides), a finding consistent with previous research [48] (Figure 2) and also significantly associated with the red and brown clusters that are enriched in the OTUs present in the AA group (Figure 3). A higher number of OTUs among *Oscillospiraceae* and *Lachnospiraceae* are enriched in AA and AI subjects and have a positive association with African foods, in agreement with Sik Novak et al. [49]. Intriguingly, the DESeq2 analysis (Figure 2) demonstrates opposing trends in certain major and minor taxa, depending on whether palm oil (composed of 50% saturated fat, 39% monounsaturated fat, and 13% polyunsaturated fat) or olive oil (composed of 15–17% saturated fat, 72% monounsaturated fat, and 8% polyunsaturated fat) was consumed. It is noteworthy that *Lachnospiraceae* UCG-003 and *Prevotella* are enriched only in the red cluster characterized by a prevalence of OTUs in the AA group (Figure 3). The minimal fluctuation observed in the AI group can be attributed to the relatively low consumption of ultra-processed foods in both the Mediterranean diet and the urban diet of Sub-Saharan Africa populations. However, the higher abundance of *Bacteroides* in the turquoise cluster compared with the red cluster could be explained by the higher consumption of fats and animal proteins together with the recent easier availability of processed foods in urban African populations, thus reducing the differences with the Italian diet across the three groups [16,29,50,51,52]. This study, in agreement with Mills et al. [53], confirms the differential effects of fats, a diverse class of macronutrients, on the Firmicutes/Bacteroides ratio. The red cluster in Figure 3 clearly illustrates this, showing a significant difference in enriched OTUs between individuals consuming palm oil and those consuming olive oil. Notably, the observed abundances of *Bilophila*, *Prevotella*, the *Christensenellaceae* R-7 group, and *Lachnospiraceae* are associated with the consumption of palm oil and products such as Cube Maggi which often include palm oil among its ingredients.

Finally, the higher relative abundances of *Escherichia–Shigella* (Proteobacteria P), considered detrimental, and the lower relative abundances of *Bifidobacterium*, considered beneficial, in the ii group (Figure 2) are not associated with the food included in this investigation. The difference may be due to factors such as gestational age, the type of delivery, breastfeeding, or environmental bacterial contamination, all of which can play a role in the different development of the microbiota in the Italian group (ii) [54].

The WGCNA (Figure 3) revealed a similar concordance between participants’ residence and relationship with food and was able to describe the diversity of the gut microbiota among the groups. The prevalence of functional predictions attributable to the AA group taxa across all four clusters, in contrast to the limited contributions from group ii and the presence of group AI only in the turquoise cluster, supports Lozupone’s hypothesis [42] that lower functional variability, compared with taxonomic variability, drives bacterial community diversity.

It is possible that the WGCNA (Figure 3), correlated with the bacterial composition of the AA group in the four clusters, is influenced by the higher presence of *Blastocystis* in this group. This protozoan may induce specific bacterial gene functions in *Blastocystis*-positive subjects, who also exhibit greater alpha and beta diversity (Figure S2 and Figure 3). However, the small sample size and the lower prevalence of *Blastocystis* in groups AI and ii highlight the need for further metabolomic investigation to validate this hypothesis.

The associations observed between food preferences and a large number of OTUs in each group are intriguing; however, given the limited sample size, further investigation is still needed to validate these preliminary findings (Figure 2 and Figure 3A).

In *Blastocystis*-negative individuals, the trend of an increased abundance of *Bacteroid*etes and reduced levels of *Prevotella* was confirmed [29,55]. Subtypes ST1, ST2, and ST3 were the most prevalent across all subjects, consistent with prior research by Cinek et al. [56]. Notably, ST4 was not detected in any African subjects (AA and AI groups), consistent with the findings of Piperni et al. [25]. In the top 20 most prevalent taxa analysis, *Blastocystis* ST3, appears to be associated with a reduced abundance of *Faecalibacterium*; however, this association was not supported by the linear discriminant analysis (LDA) (Figure 4C). Interestingly, higher *Faecalibacterium* abundance appears to correlate with the consumption of olive oil, lentils, and dried legumes, foods more common in the diet of Italian participants (Figure 2), among whom *Blastocystis* prevalence was lower and ST3 was not detected. In contrast, in a previous study, Mattiucci et al. [57] reported *Blastocystis* ST3 as the most prevalent subtype in a cohort of symptomatic Italian patients. In the present study, focusing exclusively on asymptomatic individuals, the absence of *Blastocystis* ST3 in the group of Italian individuals may be attributed to differences in clinical status rather than solely sample size limitations.

The near-statistically significant association (*p* = 0.05) between *Blastocystis* carriage and specific African food consumption (Supplementary Figure S6) points towards a potential novel factor influencing human infection that warrants further investigations. Together with genetic subtype variations, these findings suggest that diet, through its influence on the intestinal environment, may account for the increased prevalence of *Blastocystis* in asymptomatic individuals in geographical regions with a higher risk of intestinal parasite exposure.

This study has some limitations, including a small sample size, the unavailability of anthropometric data, and the diverse origins of the African individuals (group AI). However, it revealed that the gut microbiota of the AI group who lived in Italy for over 24 months continued to have significant levels of microbes typically found in African populations. This provides potentially valuable insights for developing new gut microbiota biomarkers, moving beyond the common use of the three genera: *Prevotella*, *Bacteroides*, and *Ruminococcus*.

## 5. Conclusions

This study represents the first comparative analysis of gut microbiota composition among individuals from Côte d’Ivoire, Italians, and Africans residing in Italy, linking specific, differentially abundant taxa to the consumption of common foods including palm oil, Cube Maggi, sunflower oil, and olive oil. These findings provide a valuable starting point for future comparative research on the daily intake of specific foods (e.g., olive oil and palm oil) and their effects on gut microbiota, anthropometric measures, and metabolic parameters in both Italian and African populations.

Moreover, this work contributes with novel data on the variability of the intestinal microbiota between *Blastocystis* carriers and non-carriers in Côte d’Ivoire. *Blastocystis* ST1 and ST3 carriers do not appear to exhibit a composition indicative of dysbiosis. This study may be valuable for future patient selection regarding treatment for *Blastocystis* persistence in the human gut. The relationship between foods and *Blastocystis* also highlights that positivity to eukaryote microorganisms may be one of the main confounders when comparing industrialized (virtually gut-eukaryote free) and non-industrialized populations.

## Figures and Tables

**Table 1:** Participants characteristics and detection of *Blastocystis* subtype.

African Individuals from Côte d’Ivoire				African Individuals Residing in Italy				Italian Individuals Residing in Rome			
Sample ID	SEX (7M/4F)	AGE	*Blastocystis* ST	Sample ID	SEX (5M/2F)	AGE	*Blastocystis* ST	Sample ID	SEX (7M/8F)	AGE	*Blastocystis* ST
1AA	M	37	neg	3AI	F	56	neg	1ii	F	40	neg
2AA	F	33	ST3	4AI	F	33	neg	2ii	M	51	neg
3AA	M	21	ST3	5AI	M	24	ST3	3ii	F	25	ST1
4AA	M	22	neg	6AI	M	23	neg	4ii	F	29	ST4
5AA	M	28	ST1	7AI	M	42	neg	5ii	M	20	neg
6AA	M	31	ST3	8AI	M	31	neg	6ii	F	30	neg
7AA	F	26	ST2	9AI	M	34	ST1	7ii	M	33	neg
8AA	M	22	ST1					8ii	F	36	neg
9AA	M	35	ST1					9ii	M	27	neg
10AA	F	35	neg					10ii	M	28	neg
11AA	F	44	ST3					11ii	F	26	neg
								12ii	M	27	neg
								13ii	F	40	neg
								14ii	M	31	neg
								15ii	F	26	ST4
Mean ± SD		30.4 ± 7.3				34.7 ± 11.3				31.3 ± 7.7	

**Table 2:** Percentages of subjects in each of the three groups consuming the indicated foods compared using Fisher’s Test.

FOODS	AA	AI	ii	Total
^a^ Foutou	73 (*** vs. ii)	28	0 (*** vs. AA)	33
Yam	73 (*** vs. ii)	43 (* vs. AA)	0	33
^b^ Attiéké	100 (*** vs. ii)	43 (* vs. AA)	0 (* vs. AA)	42
^c^ Cube Maggi	73 (* vs. AI)	86	0 (*** vs. AA)	42
Palm oil	75 (*** vs. ii)	0 (** vs. AA)	0	24
Olive oil	18 (*** vs. ii)	86 (* vs. AA)	100	70
Yogurt	9 (*** vs. AI)	100 (* vs. ii)	40	36
Pasta	45	100	100 (** vs. AA)	82
Potatoes	45	100	100 (** vs. AA)	82
Sunflower oil	9 (* vs. AI)	71	20 (* vs. AI)	27
Beans and lentils	3 (*** vs. ii)	12	45	54

AA indicates African subjects residing in Côte d’Ivoire, AI indicates individuals residing in Rome from different African countries, ii indicates Italian people residing in Rome. Fisher’s test significance is indicated in brackets: *** means *p* < 0.001; ** means *p* < 0.01; * means *p* < 0.05, vs. means versus. Numbers in the table indicate the percentage of subjects consuming each food item per week. ^a^ Foutou is a West African dish made by pounding boiled cassava and plantains, sometimes with palm oil. ^b^ Attiéké is made by peeling and grating cassava. The grated cassava is mixed with a small amount of previously fermented cassava as a starter and left to ferment for one or two days to reduce its hydrocyanic acid content. It is then dewatered, screened, and finally cooked by steaming. Attiéké, a food high in carbohydrates, mainly starch, is low in protein and fat. ^c^ Cube Maggi: broths produced by fermenting starch, sugar beets, sugar cane, or molasses; they contain iodized salt and some are supplemented with iron and palm oil (Supplementary Questionnaire S1).

## Data Availability

The dataset associated with this research can be accessed in the SRA database with the following identifier: PRJNA1293299 https://www.ncbi.nlm.nih.gov/sra (accessed on 18 July 2025).

## Supplementary Materials

The following supporting information can be downloaded at: https://www.mdpi.com/article/10.3390/nu17182950/s1, Figure S1: Microbial alpha diversity of three groups; Figure S2: Microbial alpha diversity in *Blastocystis* carriers; Figure S3: PCoA of the control group and *Blastocystis-*positive group; Figure S4: Microbial alpha diversity among ST1 and ST3 subtypes, and *Blastocystis* non-carrier subjects; Figure S5: PCoA of ST1 and ST3 subtypes, and *Blastocystis* non-carrier subjects; Figure S6: *Blastocystis* infection and the consumption of specific foods; Table S1: Age and sex of groups; Questionnaire S1: Food preferences questionnaire.
