# Supplementary material for: Human Gut Microbiota Profiles Related to Mediterranean and West African Diets and Association with Blastocystis Subtypes
Source: Nutrients. 2025 Sep 13;17(18):2950. doi: 10.3390/nu17182950 (PMC12472930; doi:10.3390/nu17182950)
Supplement: Supplementary file 1 [file nutrients-17-02950-s001.zip › nutrients-3797622-supplementary.pdf]

	Comparison	diff	lwr	upr	*p* adj
age	AI-AA	4.350649351	-5.74201499	14.44331369	0.544098712
age	ii-AA	0.903030303	-7.383250411	9.189311017	0.961043505
age	ii-AI	-3.447619048	-13.00263971	6.10740162	0.65097745
sex	*p*-value = 0.48				

Table S1: Age and sex of groups. No significant differences were found in age and sex among the three groups, as determined by two-way ANOVA with Tukey's post-hoc test and two-sided Fisher's Exact Test, respectively.

Collection date __________

CODE ___

AI= participants from various African countries residing in Rome- Italy
AA= participants living in Anyama-Côte d'Ivoire
ii= Italians participants living in Rome

Name ____________________________ Surname ___________ __________________

Age _________ Country of residence ____________ Country of origin __________________

***AI**: Arrival in Italy __________ □Regular returns to Africa □Last return to Italy _________

*Participants in the AI group were asked to indicate how often they ate each food per week.

Proteins: □Milk □Yogurt □Eggs □Chicken □Red meat □Fish

Carbohydrates: □Bread □Pasta □Rice □Cous Cous □Potatoes
□Foutou □Yam □Foufou □Attiéke □Kabato □Plantains □Cookies
□Snacks □Fruit juices

Fats: □Olive oil □Sunflower oil □Palm oil □Butter □Margarine

Other: □Cooked vegetables □Green beans □Dried beans □Lentils
□Other legumes □Salad □Carrots □Fresh fruit □Dried fruit □Cube Maggi

Questionnare S1: The Questionnaire assessed to the subjects of the three enrolled groups to evaluate the influence of their food preferences.

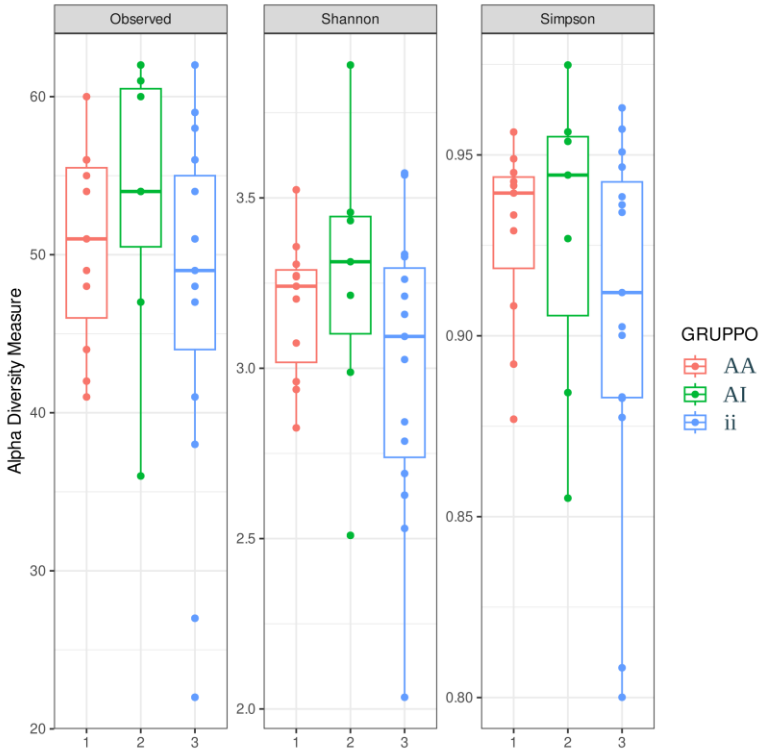

Figure S1: Microbial Alpha diversity of three groups. Among the Ivorian African group (AA), the African group residing in Rome (AI), and the Italian group (ii), Observed, Fisher and Chao1 metrics were not significant by Benjamini-Hochberg-adjusted 2-way-ANOVA.

	comparison	diff	lwr	upr	*p* adj
Obserevd	AI-AA	2.883117	-8.45346	14.21969	0.806554
Obserevd	ii-AA	-3.21212	-12.5197	6.095436	0.674863
Obserevd	ii-AI	-6.09524	-16.8279	4.63743	0.353632
Shannon	AI-AA	0.078981	-0.35691	0.514875	0.896229
Shannon	ii-AA	-0.17469	-0.53256	0.183191	0.460463
Shannon	ii-AI	-0.25367	-0.66634	0.159005	0.298178
Simpson	AI-AA	-0.00057	-0.05046	0.049313	0.999561
Simpson	ii-AA	-0.2236	-0.06331	0.018601	0.381748
Simpson	iiAI	-0.02178	-0.06901	0.025443	0.499261

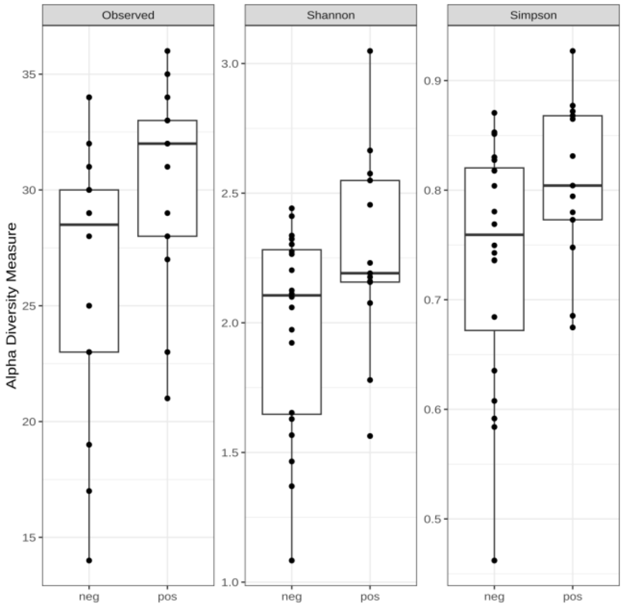

Figure S2: Microbial Alpha diversity in *Blastocystis* carriers. Analysis by two-sided Student's *t*-test of Microbial Alpha diversity by Observed, Shannon, and Simpson metrics. All *p*-values were corrected for multiple testing using the Benjamini-Hochberg criterion.

	Adj *p*
Observed	0.039899
Shannon	0.039899
Simpson	0.39899

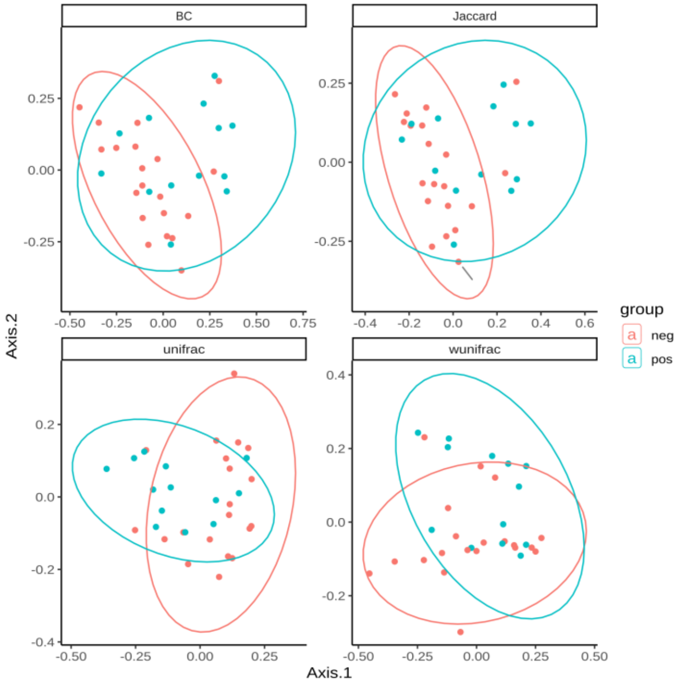

Figure S3: PCoA of the control group and *Blastocystis* positive group. PCoA of unweighted and weighted UniFrac, Bray-Curtis, and Jaccard with 95% confidence ellipses showing the significant distances between the control group and *Blastocystis* positive group. The statistical significance of sample groupings was calculated using the resulting distance matrices and the Adonis nonparametric analysis of variance. $p < 0.05$ was considered statistically significant.

	Df	SumOfSqs	R2	F	Pr(>F)
bray	1	0.4842864	0.051099765	1.669398583	0.028
jaccard	1	0.5182585	0.037868343	1.220122666	0.027
unifrac	1	0.27594487	0.056328575	1.850417173	0.005
wunifrac	1	0.19424504	0.075478378	2.530854505	0.043

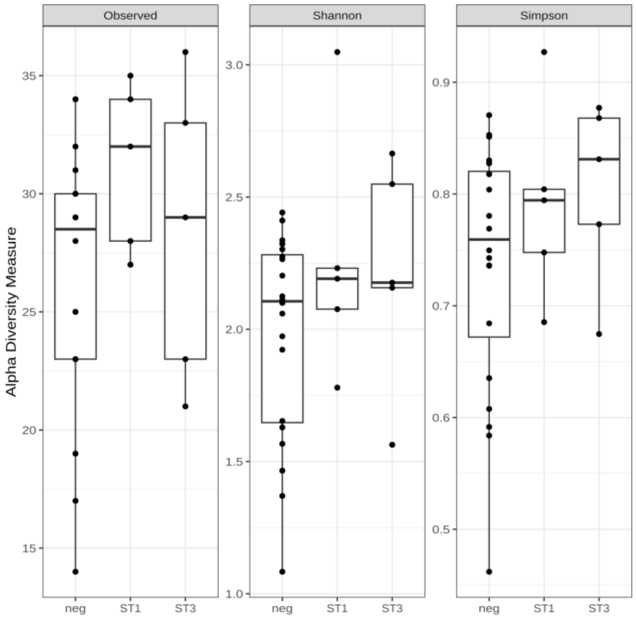

Figure S4: Microbial Alpha diversity among ST1 and ST3 subtypes, and *Blastocystis* non-carrier subjects. Microbial Alpha diversity by Observed, Shannon and Simpson metrics between *Blastocystsis* carriers and no-carriers were not significant by Benjamini-Hochberg-adjusted 2-way-ANOVA.

	comparison	diff	lwr	upr	*p* adj
Observed	ST1-neg	4.7	-1.9675048	11.3675048	0.20634409
Observed	ST3-neg	1.9	-4.7675048	8.56750476	0.76173136
Observed	ST3-ST1	-2.8	-11.233801	5.63380054	0.69214342
Shannon	ST1-neg	0.28433835	-0.2219942	0.79067086	0.35890655
Shannon	ST3-neg	0.24122821	-0.2651043	0.74756073	0.47424205
Shannon	ST3-ST1	-0.0431101	-0.835757	0.59735546	0.98477088
Simpson	ST1-neg	0.05422843	-0.0745613	0.18301816	0.55623328
Simpson	ST3-neg	0.06727049	-0.0615192	0.19606022	0.41008807
Simpson	ST3-ST1	0.01304206	-0.1498655	0.17594962	0.97853021

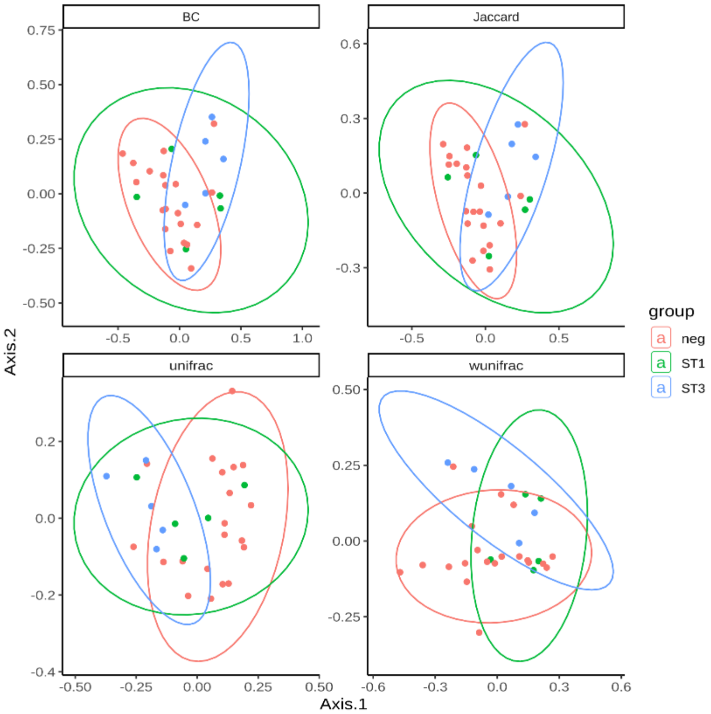

Figure S5: PCoA of ST1 and ST3 subtypes, and *Blastocystis* non-carrier subjects. PCoA of unweighted and weighted UniFrac, Bray-Curtis, and Jaccard with 95% confidence ellipses showing the significant distances among the control group and *Blastocystis* ST1 and ST3 positive groups. The statistical significance of sample groupings was calculated using the resulting distance matrices and the Adonis nonparametric analysis of variance. $p < 0.05$ was considered statistically significant.

	comparison	diff	lwr	upr	*p* adj
Observed	ST1-neg	4.7	-1.9675048	11.3675048	0.20634409
Observed	ST3-neg	1.9	-4.7675048	8.56750476	0.76173136
Observed	ST3-ST1	-2.	-11.33801	5.63380054	0.69214342
Shannon	ST1-neg	0.28433835	-0.2219942	0.79067086	0.35890655
Shannon	ST3-neg	0.24122821	-0.2651043	0.74756073	0.47424205
Shannon	ST3-ST1	-0.0431101	-0.6835757	0.59735546	0.98477088
Simpson	ST1-neg	0.05422843	-0.0745613	0.18301816	0.55623328
Simpson	ST3-neg	0.06727049	-0.0615192	0.19606022	0.41008807
Simpson	ST3-ST1	0.01304206	-0.1498655	0.17594962	0.97853021

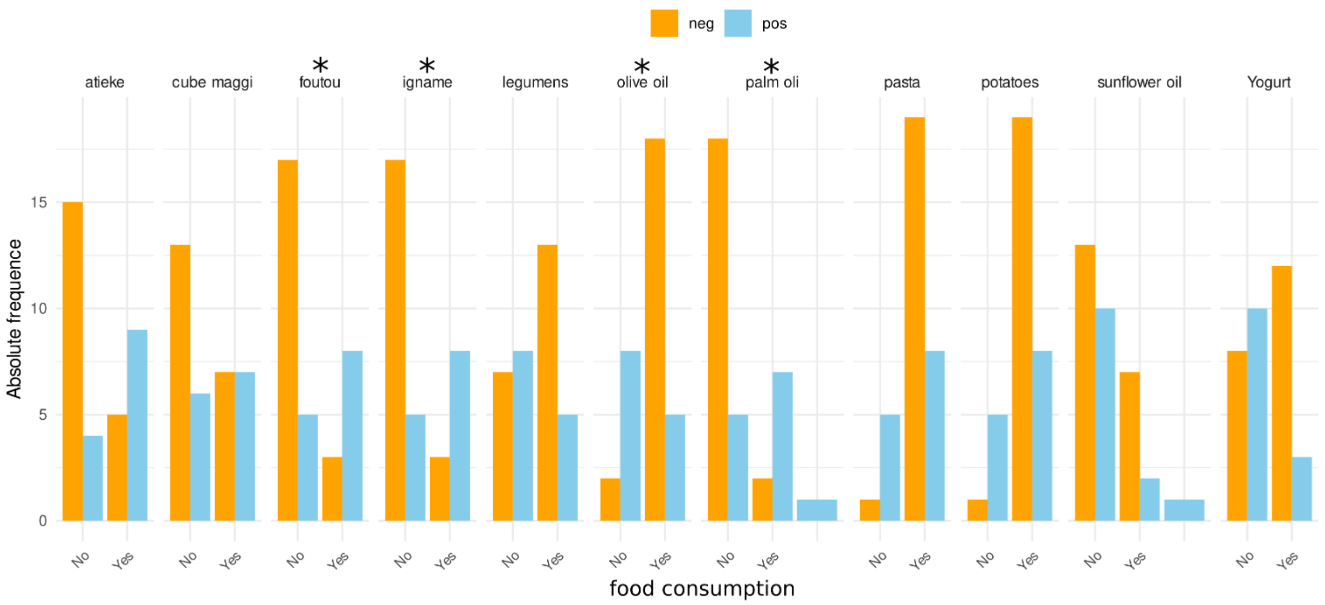

Figure S6: *Blastocystis* infection and the consumption of specific foods. The bar chart illustrates the associations between *Blastocystis* infection and the consumption of spe-cific foods by chi-squared test in *Blastocystis* carriers (sky blue) and non-carriers(orange) subjects. All *p*-values were corrected for multiple testing using the Benjamini-Hochberg criterion. * means $p < 0.05$.
